# Necrotic xanthogranuloma with disseminated annular lesions^☆^^☆☆^

**DOI:** 10.1016/j.abd.2019.03.007

**Published:** 2019-12-18

**Authors:** Isaura Azevedo Fasciani, Neusa Yuriko Sakai Valente, Maria Claudia Alves Luce, Priscila Kakizaki

**Affiliations:** a Department of Dermatology, Hospital do Servidor Público Estadual (HSPE), São Paulo, SP, Brazil; b Department of Dermatopathology, Hospital do Servidor Público Estadual (HSPE), São Paulo, SP, Brazil

Dear Editor,

Necrotic xanthogranuloma (NX) is a non-Langerhans histiocytosis, initially described in 1980,^1^ which is characterized by yellowish plaques and nodules with a tendency to ulceration, which may infiltrate mainly the periorbital region, the flexor surface of the extremities, and the trunk. There is no predilection for gender and it mainly affects middle-aged patients.

A 73-year-old man, attended the dermatology outpatient clinic, with yellowish lesions on the trunk that had benn present fortwo years. On physical examination, he showed infiltrated annular plates with clear centers and erythematous borders on the thorax and abdomen, and asymptomatic lower limbs (Figure 1, Figure 2). One of the lesions of the abdomen was ulcerated. He reported a previous diagnosis, about 20 years ago , of annular granuloma. A biopsy of the abdominal lesion was performed (Fig. 3) with the diagnostic hypotheses of necrotic xanthogranuloma, lipoidica necrobiosis, annular granuloma, and xanthoma. Histopathology showed the dermis completely compromised by a chronic granulomatous process with numerous Touton cells, some bizarre, and areas of necrobiosis with nuclear debris and collagen sclerosis. The findings favored necrobiotic xanthogranuloma. In view of this diagnosis, monoclonal gammopathy was investigated, and urinary immunofixation revealed a monoclonal band corresponding to the kappa light chain (Bence Jones) and serum immunofixation, detected by the IgG kappa monoclonal band. The patient was referred to the hematology service, where he underwent bone marrow biopsy, without criteria for hematological diseases at the time of the work up . The patient is currently using dapsone and presenting partial improvement of the lesions. He has been followed in conjunction with hematology.

**Figure 1:**
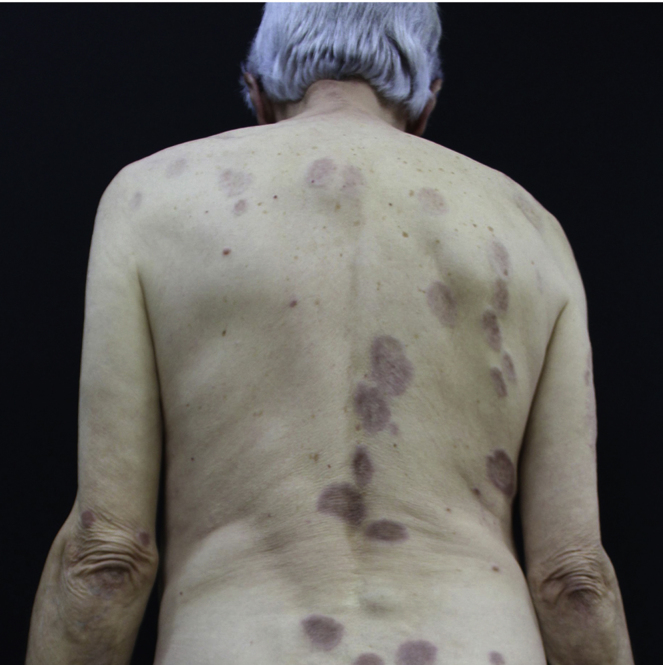
Lesions on the back. Yellowish infiltrated annular plaques with clear centers and erythematous borders.

**Figure 2:**
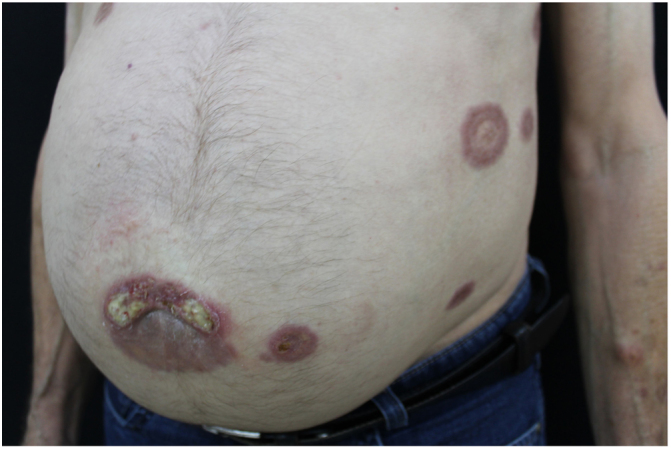
Detail of the lesion on the abdomen. Yellowish plaques on the abdomen.

**Figure 3:**
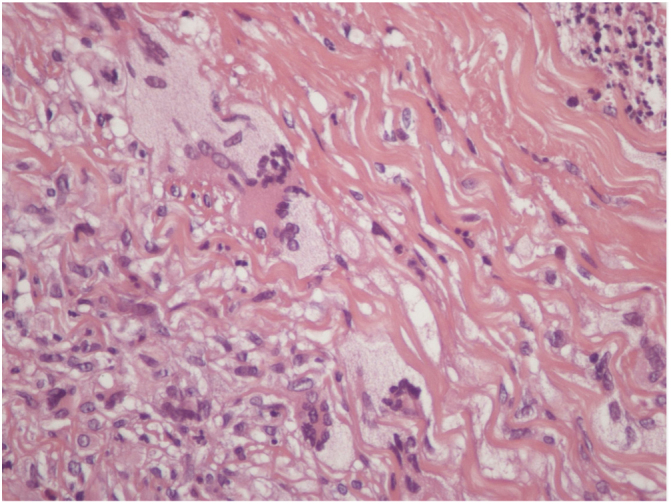
Skin biopsy. Necrotic xanthogranuloma. Presence of collagen necrobiosis and giant cells.

NX has cutaneous findings, predominantly yellowish plaques in the periorbital area, trunk, and extremities, most often associated with paraproteinemia, and can coexist with systemic involvement of multiple organs, such as the heart, respiratory system, spleen, kidneys, liver, skeletal muscle, and central nervous system.^2^ Up to 80% of patients diagnosed with NX present or will present monoclonal paraproteinemia, predominantly of the monoclonal gammopathy type IgG kappa or lambda.^1^

The association between NX and hematological disorders is well documented, with an increased risk of hematological diseases, malignancies, and lymphoproliferative disorders.^3^ Hematologic disorders may occur up to eight years before or 11 years after the appearance of cutaneous lesions.^4^ For this reason, patients diagnosed with NX require lifelong care.

Other changes that may accompany NX are neutropenia, hypocomplementemia, cryoglobulinemia, or hyperlipidemia. Associated diseases include multiple myeloma, chronic lymphocytic leukemia, Hodgkin's disease, non-Hodgkin's lymphoma, asthma, and Quincke's edema.

The differential diagnoses for NX include lipoid necrobiosis, juvenile xanthogranuloma, annular granuloma, foreign body granuloma, subcutaneous rheumatoid nodules, xanthomas (disseminated, normolipemic flat, primary, and secondary), amyloidosis, and Erdheim–Chester disease.

In histopathology, NX shows typical areas of necrobiosis surrounded by granulomas composed of giant Touton cells, foamy histiocytes, and giant foreign-type giant cells, as well as lymphocytes, compromising the entire dermis.

In the pathogenesis of NX, it is suggested that serum immunoglobulins bind to lipids, depositing in the skin, which would provoke a foreign body reaction. Another hypothesis is that paraprotein would bind the Fc portion of IgG by activating a secondary proliferation of macrophages. It has also been proposed that the paraprotein in NX has the functional characteristics of a lipoprotein that can bind to histiocyte lipoprotein receptors and induce granuloma formation.^1^ The etiology of this disorder remains obscure despite theories that attempt to clarify its pathogenesis. Consequently, treatment is difficult, without a recommended first-line therapy and a tendency to recurrent skin lesions.

Treatment options include immunomodulatory drugs, immunosuppressive agents, corticosteroids, alkylating agents, plasmapheresis, and radiotherapy.^5^ However, it has been found that even with treatment the lesions tend to be progressive, with recurrence of new lesions. Due to its rarity, there is no recommended first-line treatment. Thus, the therapeutic option should be chosen based on the hematological conditions associated with the disorder, as well as on the location, extent, and degree of impairment of the patient's life.

## Financial support

None declared.

## Authors’ contribution

Isaura Azevedo Fasciani: composition of the manuscript.

Neusa Yuriko Sakai Valente: statistical analysis, approval of the final version of the manuscript; participation in the design of the study; critical review of the literature.

Maria Claudia Alves Luce: conception and planning of the study.

Priscila Kakizaki: approval of the final version of the manuscript; participation in the design of the study; critical review of the manuscript.

## Conflicts of interest

None declared.

## References

[1] Ugurlu S., Bartley G.B., Gibson L.E. (2000). Necrobiotic xanthogranuloma: long-term outcome of ocular and systemic involvement. Am J Ophthalmol.

[2] Seastrom S., Bookout A., Hogan D.J. (2014). Necrobiotic xanthogranuloma without a monoclonal gammopathy. Cutis.

[3] Spicknall K.E., Mehregan D.A. (2009). Necrobiotic xanthogranuloma. Int J Dermatol.

[4] Wood A.J., Wagner M.V., Abbott J.J., Gibson L.E. (2009). Necrobiotic xanthogranuloma: a review of 17 cases with emphasis on clinical and pathologic correlation. Arch Dermatol.

[5] Miguel D., Lukacs J., Illing T., Elsner P. (2017). Treatment of necrobiotic xanthogranuloma – a systematic review. J Eur Acad Dermatol Venereol.

